# Mesotherapy as a Promising Alternative to Minoxidil for Androgenetic Alopecia: A Systematic Review

**DOI:** 10.7759/cureus.59705

**Published:** 2024-05-05

**Authors:** Esraa M Aledani, Harleen Kaur, Malik Kasapoglu, Rajesh Yadavalli, Sarosh Nawaz, Abdulaziz Althwanay, Tuheen Sankar Nath

**Affiliations:** 1 Dermatology, California Institute of Behavioral Neurosciences & Psychology, Fairfield, USA; 2 Internal Medicine, California Institute of Behavioral Neurosciences & Psychology, Fairfield, USA; 3 Medicine and Surgery, Maharishi Markandeshwar Institute of Medical Sciences and Research, Mullana, IND; 4 Internal Medicine, Rajiv Gandhi Institute of Medical Sciences, Adilabad, IND; 5 Psychiatry, California Institute of Behavioral Neurosciences & Psychology, Fairfield, USA; 6 Medicine, Imam Abdulrahman Bin Faisal University, Dammam, SAU; 7 Surgical Oncology, Tata Medical Center, Kolkata, IND

**Keywords:** trials, therapy, drugs, hairloss, androgenetic alopecia, alopecia, dermatology, 5% topical minoxidil, minoxidil, mesotherapy

## Abstract

Patterned hair loss (PHL) is a severe hair condition that affects both sexes. Mesotherapy is a treatment that involves microinjecting medications and/or vitamins into the middle layer of the skin. Mesotherapy reduces systemic adverse effects by delivering drugs directly to the hair follicle, increasing local bioavailability while lowering systemic exposure. Local side effects and reactions may develop due to mesotherapy. This study systematically evaluated the safety and efficacy of mesotherapy to minoxidil 5%, as well as addressing its limitations, dosing, and technique, with the intent of providing valuable trials and insights for clinicians and patients considering mesotherapy for improved androgenetic alopecia (AGA) outcomes. The literature search carried out by the Preferred Reporting Items for Systematic Reviews and Meta-Analyses (PRISMA) criteria yielded 11 relevant studies from an initial pool of 18 articles. These studies covered various aspects of the role of mesotherapy and minoxidil in AGA, including techniques, complications, limitations, and outcomes. In conclusion, available trials and research on mesotherapy and minoxidil demonstrated excellent statistical significance and a high patient satisfaction rate, with the exception of two publications that took into account certain uncommon adverse effects of mesotherapy. However, recent research suggests that a mesotherapy method for alopecia with a low risk of side effects is effective.

## Introduction and background

The advent of mesotherapy came from two combined Greek words: "meso,” which means the middle layer of the skin, and "therapy.” It involves the microinjection of medications, pharmaceuticals, and vitamins into the mesoderm. Mesotherapy was previously used by ancient European ladies to make their skin prettier [1,2]. Currently, it is used for numerous dermatological conditions. It has completely changed the field of treating androgenetic alopecia (AGA), and it is gaining lots of interest and popularity among patients. Mesotherapy involves the direct injection of the solution into the scalp, causing multiple trauma induced by the microinjection, which increases the release of cytokines and growth factors into the scalp [2].

During the Shakespeare era, people with AGA had no option but to live with it. However, in our modern era, the discovery of multiple drugs that are assumed to cure alopecia has made it crucial to actively manage the condition since it also affects our physical appearance and psychological well-being [2].

Female pattern hair loss (FPHL) is one of the most common (with a prevalence rate of 50%) and important hair problems that affects both sexes. FPHL is characterized by reduced hair density over the crown and frontal scalp (a Christmas tree pattern), with the frontal hairline remaining intact. In males, hair loss is particularly noticeable in the frontotemporal areas [2,3]. The psychological impact of FPHL on female individuals has a devastating effect, but males are more vulnerable to psychological stress due to the greater visibility of pattern baldness.

The US Food and Drug Administration (FDA) has only authorized topical minoxidil 2% and 5% for treating FPHL. However, low patient compliance and satisfaction rates, as well as a plethora of topical and, in some cases, serious systemic side effects, have prompted a quest for alternative therapeutic options [4].

Mesotherapy is derived from a Greek phrase meaning "therapy" for the skin's middle layer ("mesoderm"). It is a treatment that involves microinjecting medications and/or vitamins into the skin's middle layer [2,4]. Mesotherapy counteracts these systemic side effects by delivering the medication directly to the hair follicle, increasing local bioavailability while reducing system exposure. Mesotherapy for AGA treatment has been explored and utilized in some clinics; however, it has not been approved by the US FDA. However, mesotherapy can produce local erythema, pain, pruritus, headaches, edema, local hematoma, folliculitis, and, in rare circumstances, an inflammatory reaction, granulomatous reaction, Koebnerization, fat necrosis, and paradoxical nonscarring alopecia [5].

In this systematic review, we aim to compare the safety and efficacy of mesotherapy to minoxidil, summarize what is known from the past five years of research, and address its limitations and techniques.

## Review

Methods

Search Strategy

A PICO (Population, Intervention, Comparison, and Outcome) was formulated in this systematic review to provide a structured framework for guiding the methodology section. The patient population (P) under study comprises individuals with AGA. The primary focus of the investigation (I) is to evaluate the advantages of mesotherapy and the positive results associated with AGA. This will be compared (C) to the conventional minoxidil approach. The primary outcome (O) to be assessed is the determination of whether mesotherapy represents a viable alternative to minoxidil.

The Preferred Reporting Items for Systematic Reviews and Meta-Analyses (PRISMA) criteria were followed for this literature review [6]. The following combination of search phrases used for all databases included “mesotherapy” AND “minoxidil” AND “ alopecia.” This approach helped us identify relevant and up-to-date literature for our study. Articles were searched from PubMed, MedLine, and ScienceDirect.

The inclusion and exclusion criteria were set before screening the articles and the data extraction process, as illustrated in Table 1.

**Table 1:** The inclusion and exclusion criteria RCTs: randomized controlled trials

Inclusion Criteria	Exclusion Criteria
Peer-reviewed	Non-peer-reviewed
Targeting people with androgenetic alopecia who used either minoxidil or mesotherapy	Include other interventions
Studies that are written in English only	Studies that are written in languages other than English
RCTs, systematic reviews, and case reports	Editorials, commentaries, abstracts, and unpublished literature
Studies that have free full-text	Studies that do not have free full-text

Study Quality Assessment

Each article was analyzed individually for quality appraisal and the potential risk of bias. Three assessment tools were used to check the quality of the included papers. Systematic reviews were evaluated using the Assessment of Multiple Systematic Reviews (AMSTAR 2) [7], and a Scale for the Quality Assessment of Narrative Review Articles (SANRA) [8] was used for narrative review articles. The randomized controlled trials (RCTs) were subjected to the Cochrane risk-of-bias assessment tool scale for RCTs [9], and the case reports were assessed using the Joanna Briggs Institute (JBI) tool. The articles were scored as either high quality, low quality, or unclear, and only 70% of articles achieving the quality assessment tool were included.

Results

Study Identification and Selection

**Figure 1:**
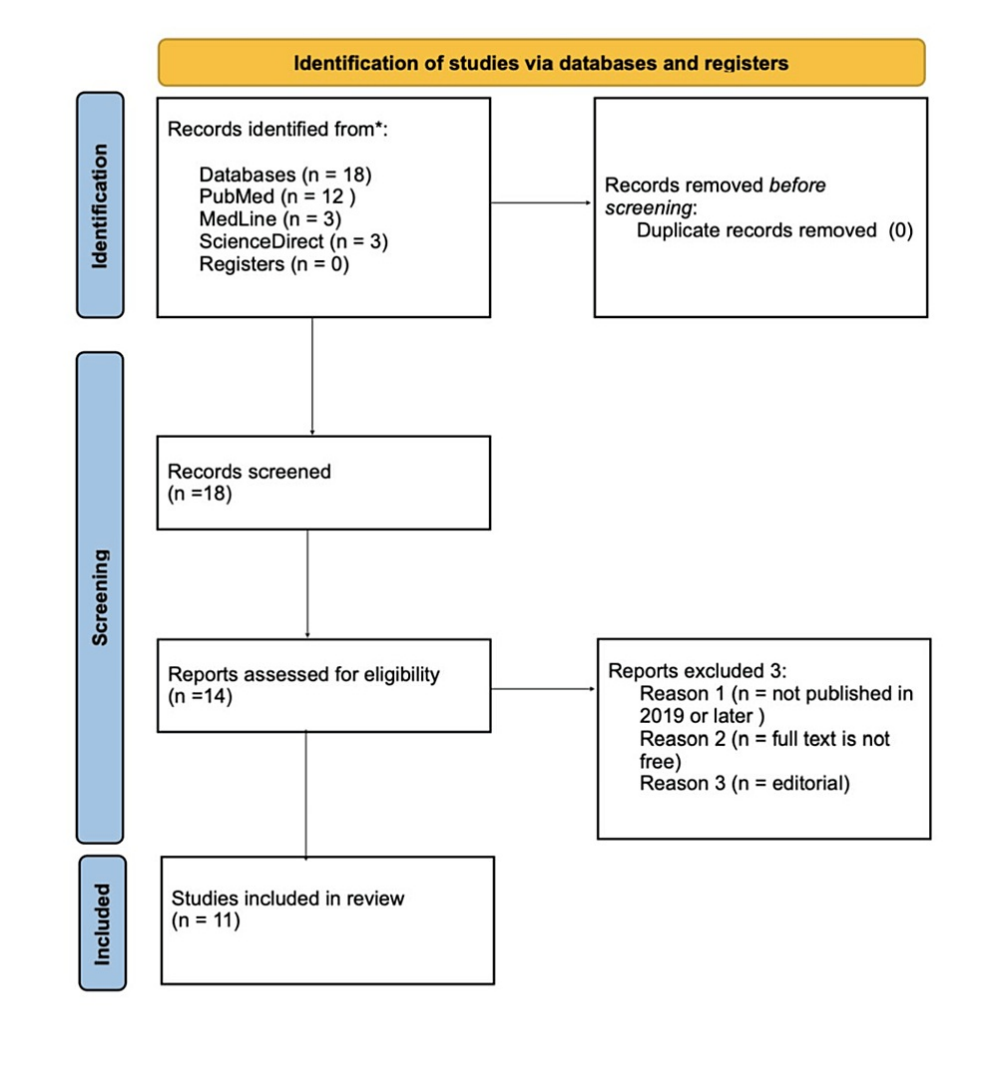
Prisma flow chart

Our systemic search yielded a total of 18 papers. After a detailed article screening, including title, abstract, and full-text screening, a total of 11 articles were included in this research, as shown in Figure 1. The ScienceDirect search engine was used to conduct a second search for articles discussing mesotherapy and minoxidil in patients with alopecia. In this review, all publications on mesotherapy and minoxidil were incorporated, and only studies that were available in the English language were included. The chosen time frame is to integrate publications featuring the most recent advancements, methodologies, and updated information, ensuring the comprehension of current knowledge and trends with accuracy and relevance. Additionally, a full-text filter was used. Out of these, six RCTs, three reviews, and two case reports and series studies were included.

The search results were transferred into EndNote and transformed into an Excel file as a second step. The duplication filter was used to eliminate the duplicate papers. After examining the article titles, relevant titles were kept on a separate sheet. The quality of these papers was then evaluated using the following scales: Cochrane risk-of-bias assessment tool for RCTs, SANRA, AMSTAR, and JBI for case reports and series. Finally, seven RCTs, two reviews, and two case reports and series studies were included.

Our systemic search yielded a total of 18 papers, and after a detailed screening, a total of 11 articles were included in this research. Out of these, a total of five RCTs, two systematic reviews, one narrative review, and three case reports and series studies were included in this study. A quality assessment check was then performed to score these papers, as shown in Table 2.

**Table 2:** Quality assessment RCT: randomized controlled trial; RoB2: version 2 of the Cochrane risk-of-bias tool for randomized trials; SANRA: Scale for the Quality Assessment of Narrative Review Articles; AMSTAR: Assessment of Multiple Systematic Reviews; JBI: Joanna Briggs Institute

Study Design	Quality Assessment Tool	Total Score	Total Score	Number of Papers
RCT	RoB2	N/A	Low risk	5
Narrative review	SANRA	12	11	1
Systematic review	AMSTAR	16	14	2
Case reports	JBI	8	8	2
Case series	JBI	10	10	1

Across all included studies, 576 patients were identified and included. All these patients had undergone either mesotherapy, minoxidil, or a combination treatment for their alopecia. More than 95% of those patients have a satisfactory result. The other 5% experienced low satisfaction due to major and minor complications, including bruising, pain, headache, frontal edema, and infection. A summary of the characteristics of all articles included is provided in Table 3.

**Table 3:** Study characteristics LLLT: low-level light-minoxidil; FPHL: female pattern hair loss; RCT: randomized controlled trial; FAGA: female androgenetic alopecia; AMSTAR: a measurement tool for the assessment of multiple systematic reviews; AGA: androgenetic alopecia; PHL: pattern hair loss; ACD: angioedema-like contact dermatitis

Study	Study Design	Number of Patients	Statistically Difference	Follow-up Period	Complications	Agent Formation	Main Outcome
Esmat et al. [1]	RCT	45	A significant difference regarding Ludwig classification was also documented in those who received combination therapy (group C) (P=0.005).	4 months	Irritation, scalp tenderness.	Low-level light-minoxidil (LLLT) in comparison to topical minoxidil 5% and to a combination of both therapies.	In the treatment of FPHL, low-level light-minoxidil 5% is an effective and safe method with equivalent outcomes to minoxidil 5%. Combination treatment is indicated to speed hair regeneration due to its much greater benefits.
Gajjar et al. [2]	RCT	49	No statistically significant difference between mesotherapy and minoxidil.	4 months	Erythema, headache.	Mesohair solution had 56 constituents, which included 24 amino acids, 13 vitamins, four coenzymes, four nucleic acids, five minerals, and two reducing agents. Decapeptide 4, acetyl decapeptide, and copper tripeptide were the active ingredients.	More research with a larger sample size and a longer follow-up time are needed to determine the effect of mesotherapy in AGA. Although effective therapy choices are limited, AGA continues to be a field where ongoing research is contributing more understanding about pathophysiology and more therapeutic alternatives are being developed accordingly.
Lucky et al. [3]	RCT	381	N/A	48 weeks	Pruritus, dermatitis, hypertrichosis, scaling, headache.	5% and 2% topical minoxidil solutions.	Superiority of 5% topical minoxidil over placebo and 2% topical minoxidil with promoting hair growth.
Hunter et al. [4]	RCT	30	A statistically significant difference was found between groups regarding the increase in the number of hair follicle after treatment, with the mesotherapy group showing more increase. There was no significant difference between the groups regarding the change in the diameter of the largest hair follicle after treatment.	12 weeks	Headache, pain.	Amino acids (alanine, arginine, aspartic acid, cystine, glutamine, glycine, histidine, isoleucine, leucine, lysine, phenylalanine, proline, serine, taurine, and threonine), minerals (zinc, selenium, copper, manganese, and chrome), hyaluronic acid (1.5%), ginkgo biloba extract, and vitamins (A, C, E, and B-complex).	In the treatment of FPHL, mesotherapy with vitamins and minerals alone is more successful, patient-acceptable, and bearable than topical minoxidil 5%.
Alhanshali et al. [5]	Narrative review	N/A	N/A	N/A	N/A	N/A	Current research suggests that mesotherapy may be a successful treatment method for alopecia with a minimal risk of side effects, although it has the limitations that have been highlighted.
Cura et al. [10]	Case report	2 women	N/A	3 months	Injection-site infections, granulomatous foreign body reactions, fat necrosis, lichenoid drug eruptions, and Nicolau syndrome, pain, headache, itching.	Mesotherapy dutasteride (second-generation 5a-reductase enzyme inhibitor that decreases serum dihydrotestosterone levels by 90%).	Two cases of paradoxical nonscarring alopecia following dutasteride mesotherapy were described. In both cases, ethanol was utilized as a solvent in a solution containing dutasteride. Based on the current ethanol toxicity data, we believe that this drug may cause cell damage and death in the hair follicle, resulting in hair loss.
Tang et al. [11]	Systematic review	N/A	N/A	N/A	N/A	Mesotherapy	Mesotherapy has shown some positive results in treating PHL without significant side effects, and it is regarded as a potential treatment for hair loss and recommended as a List B procedure in a guideline for doctors working in aesthetic practices when approved treatments fail to provide an ideal outcome. Although it is still in its early stages and has challenges in terms of popularization in hair regrowth, a better use of mesotherapy in PHL may be anticipated with clear instructions and suggestions.
Gupta et al. [12]	Systematic review	N/A	N/A	N/A	Headache, injection-site pain, and scalp tightness or itching.	Mesotherapy	Mesotherapy is a method of intradermal medication and bioactive material delivery with the potential to cure hair loss diseases such as PHL and TE. Several studies have found statistically significant increases in hair growth following mesotherapy injections with various therapeutic agents and homoeopathic solutions. Lower medication dosages, targeted delivery, and fewer injections are all aspects that can increase the usefulness of mesotherapy.
Melo et al. [13]	Case series	14	N/A	4 days	Burning, erythema and headaches, subcutaneous necrosis, scalp abscesses, and angioedema, frontal edema.	Mesotherapy	This is the largest case series focusing on frontal edema after AGA mesotherapy and provides doctors with useful information while doing this method. More research is needed to investigate the function of minoxidil, dutasteride, and possibly lidocaine as potential causes of frontal edema in mesotherapy.
Magdaleno-Tapial et al. [14]	Case report	1	N/A	24 hours	Facial swelling and erythema.	Mesotherapy with dutasteride.	Mesotherapy is a popular cosmetic technique, and ACD has already been mentioned as a side effect. This is the first report of ACD induced by dutasteride. More research is needed to determine the appropriate dutasteride patch test concentration.
Uzel et al. [15]	RCT	54	N/A	10 weeks	Frontal edema, mild pain and burning sensation, multifocal scalp abscess.	2 mL of 0.5% minoxidil against 2 mL of 0.9% saline.	Intradermal injections of 0.5% minoxidil solution once a week for 10 weeks were shown to be more effective than placebo in the treatment of FAGA, with no significant side effects.

Discussion

While certain studies show significant improvement in alopecia patients who undergo mesotherapy injections, there are growing concerns among physicians and some patients regarding its safety and efficacy due to reported complications such as angioedema and frontal edema. This article compares the efficacy and safety of mesotherapy and minoxidil, as well as its implications in dermatological practice.

There are very limited resources on using mesotherapy on the scalp for alopecia. The RCTs conducted by Gajjar et al. [2] and Hunter et al. [4] included a total of 95 patients (65 and 30 patients, respectively). Each study divided its participants into two groups. Group A was a control group that used minoxidil, and Group B used topical mesotherapy 5%. In Gajjar et al.'s [2] study, the follow-up period was four months, and the injection was done weekly, while in Hunter et al.'s [4] study, the injection was performed once weekly with 2 mL of mesotherapy for 12 weeks. The study results showed no statistically significant difference between the two groups. The patients were moderately satisfied, and the patients using mesotherapy showed improvements up to 25%-50%. A statistically significant relationship was also observed between the post-treatment diameter and the number of hair follicles (P=0.001). However, the study from Egypt showed some significant side effects in the minoxidil group, including hypertrichosis of the face, itching, and scaling. In contrast, the mesotherapy group reported headache and pain as side effects.

However, the study from India revealed that the mesotherapy group experienced mild side effects, specifically erythema in 88% of cases. On the other hand, patients using minoxidil complained of headaches in 17% of cases, as well as erythema. These findings suggest that mesotherapy is a safe treatment option. These studies focused on the comparative effectiveness of mesotherapy and minoxidil and which one was more statistically significant than the other, as well as evaluating its safety and tolerability. These studies have some limitations, including smaller sample sizes, patients being lost during follow-up, and shorter follow-up periods. Therefore, our systematic review aims to collect all the studies, including mesotherapy for alopecia, and increase the sample size by comparing and contrasting studies to achieve more accuracy and efficacy in using mesotherapy for AGA [2,4].

Alhanshali et al. [5] conducted a narrative review on the effectiveness of dutasteride mesotherapy (administering 1-2 mL of 0.05% solution) over saline mesotherapy with low-rate complications such as local pain, pruritus, headache, and ecchymosis. These complications typically resolve within 24 hours with no sexual side effects such as decreased semen volume, concentration, motility, and morphology. Furthermore, two patients had scarring alopecia after receiving dutasteride mesotherapy due to ethanol. However, further study is needed while utilizing dutasteride [5].

The study by Cura et al. [10] raises concerns about bias and challenges associated with dutasteride mesotherapy. Two examples of nonscarring alopecia in women were addressed following the use of dutasteride mesotherapy 0.005%. This outcome is thought to be caused by either a delayed cutaneous reaction to mesotherapy or a sudden reaction to manufacturing changes in the composition of the injected fluid. Dutasteride, a hydrophobic drug, must be dissolved in ethanol or dimethyl sulfoxide (DMSO). Ethanol promotes cell death, followed by increased reactive oxygen species formation, autophagy activation, and nuclear factor kappa B translocation. Indeed, given the known involvement of ethanol in causing cell death, it is now being studied as a potential medication for accelerating tumor cell death in prostate cancer and hepatocellular carcinoma. Based on our findings, we believe that the toxicity of the ethanol used in the formulation contributed to hair follicle damage. However, fewer than 10 instances were recorded with concerns; the most common problems were headache, itching, discomfort, injection-site infections, fat necrosis, granulomatous foreign body responses, lichenoid drug eruptions, and Nicolau syndrome [10].

According to Tang et al. [11] and Gupta et al. [12], there are five particular approaches for mesotherapy injection to prevent musculoskeletal strain caused by doing many injections in one place, with gauges 28-30 and 4 mm long, or with mesogen lasting 10-30 minutes. These five procedures differ in needle depth and/or angle of injection, which are as follows: (1) The intra-epidermal technique (depth: 1 mm) involves injecting multiple small amounts of the mesotherapy solution into the epidermis, often in a grid-like pattern 1 cm apart, covering the entire treatment area; each injection is applied with light pressure, causing minimal pain and no bleeding, making this technique suitable for patients with a low pain tolerance threshold. (2) The papular formation technique (depth: 2-4 mm) injects mesotherapy fluid at the dermo-epidermal interface, producing a small papule to form, and is used to treat alopecia or wrinkles. (3) The nappage technique (depth: 2-4 mm) entails multiple superficial injections at a 30-60° angle, with constant pressure applied to each injection, introducing one drop of mesotherapy solution per site; this technique is widely used for scalp treatments and skin rejuvenation, but it may cause more patient discomfort than other techniques. (4) The point-by-point technique (depth: 4 mm) involves perpendicular deep injections of 0.02-0.05 mL mesotherapy solutions into the dermis, spaced 1-2 cm apart. (5) The microperfusion approach includes injecting the same mesotherapy fluid over 10 minutes. The sleeping and point-by-point techniques are the most commonly used for mesotherapy hair loss treatment [11,12].

The advantages of utilizing a mesogun versus gauges 28-30 injection include faster injection, more exact dosage distribution, and constant depth insertion, making it a preferable alternative. Future developments may resemble microneedles, which allow for several injections in a short amount of time. However, in order to alleviate patient discomfort, several needleless procedures have been documented that may remove skin reactions while maintaining the same level of medicine penetration. This includes the use of low-power ultrasound (20-25 kHz), which may increase skin permeability by generating microscopic gas cavities, and iontophoresis, which improves transdermal medicine delivery by applying an electric current (0.5 mA/cm^2^) [12].

In comparison, the case series of Melo et al. [13] and Magdaleno-Tapial et al. [14], which included 15 patients in total, focused on the complications and limitations associated with using mesotherapy. Magdaleno-Tapial et al. [14] discussed the case of a 45-year-old lady who had significant facial edema and erythema 24 hours after her first mesotherapy with dutasteride for AGA. Angioedema-like contact dermatitis (ACD) is a condition misdiagnosed as angioedema. Androgens are responsible for reducing bradykinin synthesis and enhancing its breakdown, a powerful vasodilator that increases vascular permeability and can cause angioedema. Dutasteride's antiandrogenic properties may have a role in bradykinin buildup. Mesotherapy is a cosmetic technique that is becoming more popular, and ACD has already been identified as a side effect. This was the first case of ACD induced by dutasteride. Further research is needed to determine the appropriate patch test concentration of dutasteride.

Similarly, a case series conducted by Melo et al. [13] reported 14 women with serious side effects after undergoing mesotherapy for AGA. Half of the patients reported tenderness, followed by pain and a burning sensation. Additionally, two patients developed frontal edema after the first two sessions, which lasted for four days. However, it is important to note that the US FDA has not approved mesotherapy as a safe and effective procedure, although the FDA has approved the drugs used in the injections. Therefore, further studies are still necessary to investigate this matter [13,14].

Esmat et al. [1] and Lucky et al. [3] conducted two RCTs to assess the safety and effectiveness of minoxidil. Esmat et al. [1] conducted a four-month research with three days of therapy each week, comparing low-level light-minoxidil (LLLT) to topical minoxidil 5% and to a combination of 45 female patients with AGA. Patients were separated into three groups based on Ludwig categorization. Group C (Combined) showed considerably superior outcomes (P=0.001) and had the most significant level of patient satisfaction (P=0.027). Except for minor discomfort, no significant adverse effects were found. In a 48-week research with 381 women, Lucky et al. [3] evaluated the effectiveness and safety of 5% topical minoxidil to 2% topical minoxidil and placebo in AGA. The study found that 5% topical minoxidil outperformed placebo and 2% topical minoxidil in promoting hair growth. However, the 5% topical minoxidil group experienced more dermatological adverse effects (pruritus, dermatitis, hypertrichosis, scaling) than the 2% topical minoxidil group [3].

Furthermore, Uzel et al. [15] conducted an RCT to compare the safety and effectiveness of mesotherapy with 2 mL of 0.5% minoxidil with 2 mL of 0.9% saline in 54 female AGA patients during a 10-week period. The study found that hair density decreased in the control group and increased in the experimental group six weeks after the final injections, albeit not statistically significant (P=.054). Regarding self-assessment, 69.2% of patients in the experimental group and 37.5% in the control group reported improved hair loss (P=.028). Although the individuals did not exhibit hypertrichosis, they experienced self-limited pain, pruritus, and burning, which was more severe in the mesotherapy group (P<0.10). One disadvantage of the minoxidil mesotherapy study is the lack of a comparison with other minoxidil dosages and concentrations, such as low-dose oral minoxidil (LDOM) and topical minoxidil 5%, which have proven to be more effective than topical 2% minoxidil and are often used nowadays [15].

Limitations

The limitations of the current study included the lack of a sufficient number of studies considering mesotherapy as a hair loss treatment for more accurate outcomes, as well as limitations with the included studies, such as low participant numbers, short treatment duration, and high medication costs. Additionally, we limited our selection of publications to those written in English and available in full-text for free.

## Conclusions

Studies on mesotherapy and minoxidil showed good statistical significance and a high patient satisfaction rate, with the exception of two papers that reported rare side effects associated with mesotherapy. However, the current studies suggest the effectiveness of a mesotherapy strategy for alopecia with a low rate of side effects. More extensive studies need to be established to determine the safety and efficacy of mesotherapy for AGA treatment and to provide more perspective about the optimal use of mesotherapy in a larger control group.
